# UNGASS 2016 on drugs, the first challenge for HIV advocates in the post-2015 era

**DOI:** 10.7448/IAS.17.1.19274

**Published:** 2014-07-21

**Authors:** Ann Fordham, Khalid Tinasti

**Affiliations:** 1 International Drug Policy Consortium, London, United Kingdom; 2 Global Commission on Drug Policy, Geneva, Switzerland

**Keywords:** people who inject drugs, drug policy, United Nations General Assembly Special Session, HIV/AIDS, hepatitis C, post-2015 development agenda, harm reduction resourcing, Advocacy

Two important global policy processes are underway that will shape the future of the HIV response for people who inject drugs (PWID). The first is the debate around the post-2015 development agenda that is currently in play at United Nations in New York, where member states, HIV advocates, communities, scientists, policymakers, healthcare providers and donors are discussing a specific target towards ending the AIDS epidemic. The current MDG target, to reduce new HIV infections among PWID by 50% by 2015 [1], has sadly not been achieved. A new target should be included that focuses on the decline of HIV incidence, the reduction of stigma against key-affected populations, improved access to antiretroviral treatment and the end of AIDS-related deaths. The second process, for which momentum is building among drug policy advocates, networks of people who use drugs (PWUD), policymakers, scientists, academics and UN agencies, is the preparation for the UN General Assembly Special Session (UNGASS) on the “World Drug Problem” to be held in 2016 [2]. These two processes, almost happening in parallel, are complementary. The post-2015 development goal on health will determine the global HIV response architecture for the coming decades, while the UNGASS on drugs will define the global policy environment in which the HIV response for PWID will operate. Both processes require vigilant and sustained advocacy from civil society and community organizations working in the field of HIV and drug use.

Within the process of negotiations around the post-2015 Sustainable Development Goals (SDGs), a deeply disturbing target temporarily appeared in the “zero draft” [3] – to “eliminate narcotic drug and substance abuse” by 2030. The process by which the target was included was questionable, with no member states taking responsibility for inserting this language into the draft. Calls for this language to be removed have succeeded, but another problematic target around drug use remains which reads, “strengthen prevention and treatment of narcotic drug and substance abuse.” There is a general consensus among drug policy advocates that drug use per se is not an issue for the post-2015 development agenda, although if the overarching goal is to “attain a healthy life for all” (proposed goal 3) the most relevant target would be to improve coverage of harm reduction services. However, some advocates propose that the upcoming UNGASS is the right forum for a debate on an evidence-based and appropriate response to drug use.

The UNGASS will be a pivotal moment in the drug policy debate. It is being convened by the UN General Assembly following an urgent request from the presidents of Mexico, Colombia and Guatemala to review current drug policies and consider alternative approaches [2]. These governments are no longer willing to bear the high human and economic costs of fighting the drug trade given that these efforts have failed to significantly reduce the scale of the drug market and have led to a myriad of severe negative consequences. Ninety-five other countries signed on to the UN resolution [4] calling for the Special Session. This reflects growing concerns regarding the impacts of the current international drug control system on health (including HIV, other infectious diseases and non-communicable diseases), development, human rights, human security, poverty, migration and sustainable livelihoods.

PWID represent up to 10% of people living with HIV globally [5]. This number varies regionally from an estimated 5% in Eastern Europe to a staggering 28% in Asia [5]. Three million people, out of the 15 to 16 million PWID, are living with HIV [6]. Furthermore, an estimated 60% [7] of PWID live with the hepatitis C virus, which is a severe epidemic among this population, and a “growing public health, social and economic burden” [7].

An overly punitive global drug control framework reinforces zero-tolerance approaches to drug use and justifies repressive measures such as criminalization and compulsory rehabilitation. Stigma and discrimination, fear of arbitrary arrest, police harassment and imprisonment drive people away from health services and deter people from accessing life-saving harm reduction services, such as needle and syringe programmes, which in turn is exacerbating HIV and hepatitis C risks [8]. The woeful lack of HIV prevention in prisons and closed settings further undermines HIV outcomes for PWID as “incarceration has been associated with syringe sharing, unprotected sex and HIV outbreaks in many places around the world” [8]. Compulsory drug detention, still a widespread practice in South East Asia, has been condemned by the UN [9] and a number of donor agencies as a serious violation of human rights which in no way constitutes “drug treatment” [10].

This punitive approach to drug use also leads to a severe under-resourcing of harm reduction programmes. The UNAIDS Investment Framework estimates that USD 2.3 billion will be needed in 2015 to fully fund the HIV response for PWID (falling to USD 1.5 billion by 2020 as a result of the averted HIV infections) [11]. If just a fraction of the USD 100 billion spent on drug law enforcement [12] could be diverted to protecting the health and human rights of PWID, we would have a fighting chance of bucking the trend. Global harm reduction advocates have begun to call for governments to spend just 10 cents, out of every USD 1 currently spent on drug law enforcement, on health, development and human rights to have a real and sustained impact on the HIV and hepatitis C epidemics among PWID.

The UNGASS in 2016 will be an important moment to demand that drug policies be truly underpinned by public health and human rights. Governments can no longer claim that repressive measures will result in lower rates of drug use [13], and they must acknowledge the evidence that the decriminalization of drug use has not led to significant increases in consumption [8]. There is enough flexibility within the UN drug conventions to reform harsh drug laws. This flexibility must be exploited to the fullest extent so that PWUD are no longer subjected to punishment and repression. Harm reduction programmes must be sustainably resourced and scaled up.

And as new HIV targets are set under the post-2015 development agenda, these changes in policy and in the funding environment must be enacted to ensure that these new targets are not sadly missed as well.

The year 2016 will be important for the HIV response more broadly given that a high-level meeting on HIV/AIDS has just been announced by the UN General Assembly [14]. To ensure robust and visible civil society voices on these issues and in these important fora, drug policy reform advocates will need the support of HIV activists and vice versa, because a key underlying factor of sustainable and impactful harm reduction programmes is a supportive policy and legislative environment. Drug policy reform is a crucial step towards ending AIDS.
